# Gene coexpression network analysis identified potential biomarkers in gestational diabetes mellitus progression

**DOI:** 10.1002/mgg3.515

**Published:** 2018-11-25

**Authors:** Xiaomin Zhao, Wen Li

**Affiliations:** ^1^ Department of Obstetrics Tianjin Central Obstetrics and Gynecology Hospital Tianjin China

**Keywords:** GDM, gene module, GEO, GSEA, WGCNA

## Abstract

**Background:**

Gestational diabetes mellitus (GDM) is one of the most common problems during pregnancy. Lack of international consistent diagnostic procedures has limit improvement of current therapeutic effectiveness. Here, we aimed to screen potential gene biomarkers that might play vital roles in GDM progression for assistance of its diagnostic and treatment.

**Methods:**

Gene expression profiles in four GDM placentae at first trimester, four GDM placentae at second trimester, and four normal placentae were obtained from the publicly available Gene Expression Omnibus (GEO). Weighted gene coexpression network analysis (WGCNA) indicated two gene modules, that is, black and brown module, that was significantly positively and negatively correlated with GDM progression time points, respectively. Additionally, a significant positive correlation between module membership (MM) and degree in protein–protein interaction network of brown module genes was observed.

**Results:**

*KIF2C*, *CENPE*, *CCNA2*, *AURKB*, *MAD2L1*, *CCNB2*, *CDC20*, *PLK1*, *CCNB1,* and *CDK1* all have degree larger than 50 and MM larger than 0.9, so they might be valuable biomarkers in GDM. Gene set enrichment analysis inferred tight relations between carbohydrate metabolism or steroid biosynthesis‐related processes and GDM progression.

**Conclusions:**

All in all, our study should provide several novel references for GDM diagnosis and therapeutic.

## 1 INTRODUCTION

Gestational diabetes mellitus (GDM) represents the most common pathoglycemia form in pregnant women that might also cause hypertension and cardiovascular disease (Oliveira et al., 2015). GDM's morbidity is about 2%–5% across worldwide (Ashwal & Hod, 2015), and which is deeply affected by pathological and environment factors. For example, dangerousness of GDM could be increased up to 3.8 times in obese pregnant women than those with normal body mass index (Pantham & Aye, 2015). Previous study even reported that social capital, such as neighborhood trust, emotional support, has obvious influences on prevalence of GDM (Mizuno et al., 2016). What is more, GDM could increase incidence of type 2 diabetes mellitus and obesity in not only women, but also their offsprings (Catalano, 2010; Coustan, 2013; Harreiter, Dovjak, & Kautzky‐Willer, 2014; Nikolic et al., 2016).

Gene expression profiling has been an important mean for exploring disease mechanism and identifying valuable diagnostic and treatment biomarkers particularly after the development of gene microarray and high‐throughput sequencing technologies (Janikova et al., 2011; Kanda et al., 2015; Vu et al., 2015). As a polygenic disease, GDM progression was generally considered to be promoted by aberrant expression of multiple genes in a gestational age‐dependent manner (Uuskula et al., 2012). Insulin has been a key agent with effective treatment results for GDM mainly through regulating cholesterol transport in human placenta (Dube & Ethier‐Chiasson, 2013). Differences exist in placenta surfaces that exposed to bloodstreams of mother and fetus which corresponding to trophoblasts and endothelial cells, respectively, and could yield large amount of insulin receptor. Through interacting with those receptors, insulin could strikingly control placental gene expression shifts from mother to fetus over the time course of pregnancy, which might shed new light on exploring therapeutic targets for GDM (Hiden et al., 2006). It was also previously reported that reduced trophoblast apoptosis along with elevated inflammation could significantly result in aberrant expression of associated placental transcripts or proteins followed by GDM‐related increased placenta and newborn weights (Magee et al., 2014).

Genetic variation including single nucleotide polymorphisms (SNPs) and structure variation makes up the most common variations across the human genome, and it has been applied for multiple disease diagnostics and treatments. Vitamin D is closely associated with β‐cell function and impaired glucose absorption in GDM through transportation to placenta by vitamin D‐binding protein, that is, vitamin D receptor, and SNPs in which have been previously reported to be correlated with GDM clinical parameters (Wang, Wang, et al., 2015). Several previous studies even developed SNP‐based risk score for GDM prediction. For example, through including type 2 diabetes‐related risk variants, Kawai et al. (2017) developed a risk score formula that could stably predict GDM risk; Chawla et al. (2014) proposed a model comprised of 48 SNPs along with several common clinicopathological features including ancestry, sex, gestational age, and so on, that could improve prediction of large‐for‐gestational‐age and newborn adiposity. Although the recent advancement of understanding about GDM mechanisms, the efficacy of conventional therapy is still poor and further studies for identifying valuable biomarkers are still needed.

In this study, we conducted weighted gene coexpression network analysis (WGCNA) for time series gene expression profiles in GDM samples at first trimester and second trimester as well as control samples. Compared with traditional methodologies that take every transcript in the microarray alone and only capture two few information than that the microarray could provide, WGCNA takes correlations among those transcripts into account and identified potential disease‐related gene coexpression modules (GCMs) by considering associations between GCMs and disease's traits as well as intramodular associations. We identified several gene modules that closely associated with GDM progression and screened potential biomarkers via combination with protein–protein interaction (PPI) network. Additionally, carbohydrate metabolism or steroid biosynthesis‐related pathways were obtained through gene set enrichment analysis (GSEA), which has been previously reported to associate with GDM development.

## 2 MATERIALS AND METHODS

### 2.1 Gene expression profile dataset

We searched the Gene Expression Omnibus (GEO) with the keywords of “(gestational diabetes mellitus)” AND “Homo sapiens[porgn:__txid9606]” and only retained gene expression datasets that profiled by using Affymetrix HG‐U133 Puls2.0 platform (ref no.: GPL570). As a result, we obtained one dataset that deposited by Mikheev et al. (2008) with the accession number of GSE9984, which consisted of placenta expression profiles of four GDM patients at first trimester (45–59 days), four GDM patients at second trimester and four control samples.

### 2.2 Gene expression preprocessing

*R* and *Bioconductor* packages were applied for preprocessing raw gene expression profiles. Background correction, normalization, and logarithm transformation were conducted by using the *affy* package (Gautier, Cope, Bolstad, & Irizarry, 2004). Probes were annotated as gene symbols based on the GPL570 annotation file, and average expression value was used for genes annotated by multiple probes.

### 2.3 Weighted gene coexpression network analysis

Gene coexpression analysis is a powerful mean for exploring correlations among genes at specific conditions, particularly in time series analysis. Weighted gene coexpression network analysis (WGCNA) assigns a connection weight to each gene pair in the coexpression network and uses soft thresholds that should be more biologically meaningful compares with traditional methods that use binary information (0 = unconnected, 1 = connected) (Zhang & Horvath, 2005). *WGCNA* package is a collection of *R* functions for soft power selection, GCM detection, identification of module eigengene (first principle component of the module), assessment of associations between modules and clinical traits, and so on (Langfelder, 2008). Here, we screened GCM based on gene expression profiles of the 12 samples and assessed associations between modules and GDM by using pregnancy age as clinical variable.

### 2.4 Protein–protein interaction analysis

Protein–protein interactions among genes were obtained from the STRING database (Szklarczyk et al., 2015), containing interactions that with reliability scores according to means by which they were presented, such as high‐throughput, bioinformatics prediction, or low‐throughput methods. Here, we used reliability score >0.9 as the threshold for screening of valuable gene pairs. Cytoscape version3.6.0 (Su, Morris, Demchak, & Bader, 2014) was applied for visualizing PPI network.

### 2.5 Gene set enrichment analysis

Gene set enrichment analysis was performed by using the GSEA software (Subramanian, Kuehn, Gould, Tamayo, & Mesirov, 2007). “C2.CP.V6.0.ENTREZ.gmt” was used as the gene set for the analysis. Permutation number was set to 1,000, and *p* value <0.05 was considered as statistically significant.

## 3 RESULTS

### 3.1 Gene coexpression modules

**Figure 1:**
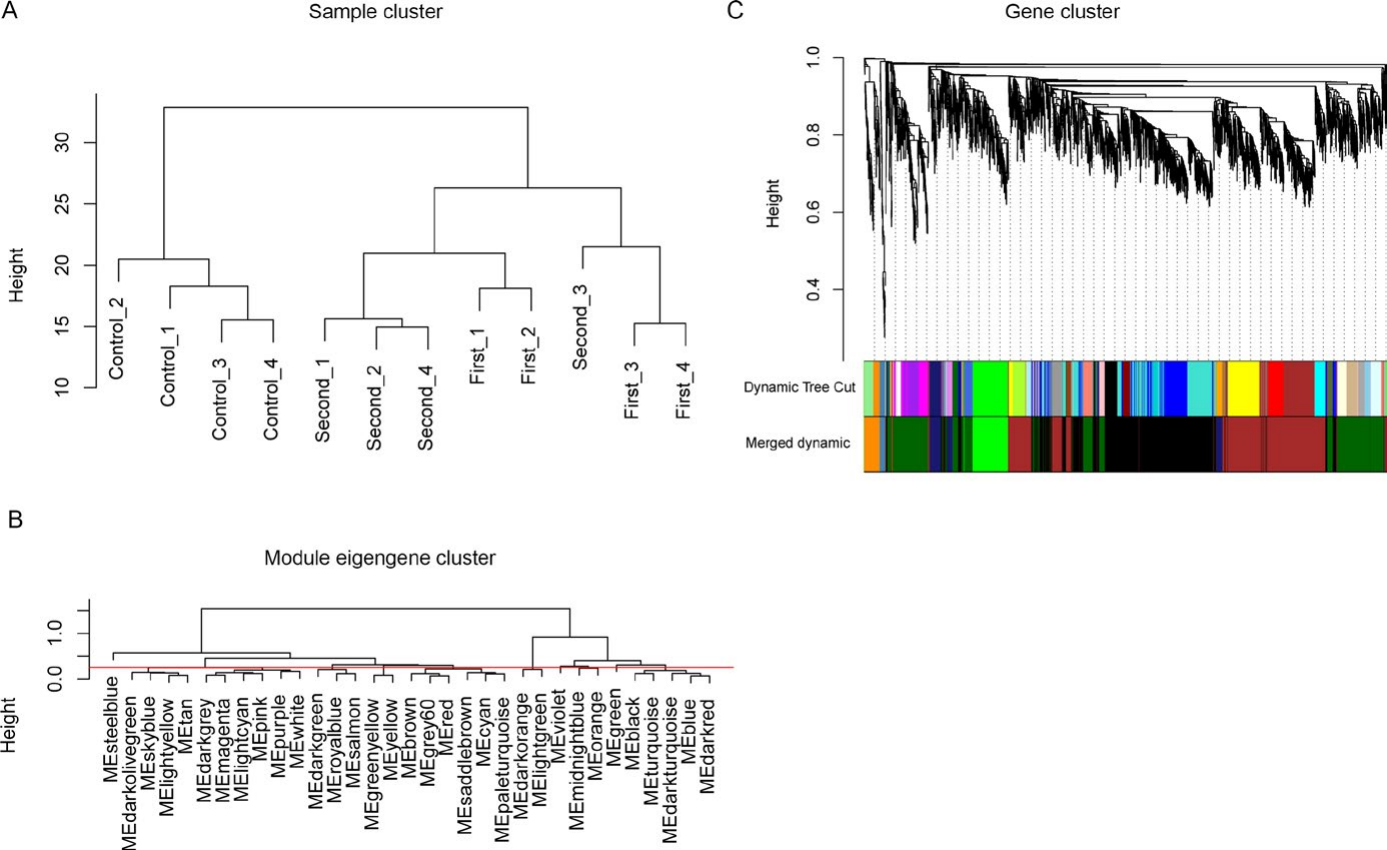
Sample and gene clustering analysis based on the 3,731 gene expression profiles. (a) Sample clustering identified two main clusters that containing gestational diabetes mellitus and control samples, respectively. (b) Gene coexpression modules (GCMs) obtained through weighted gene coexpression network analysis and GCM clusters based on their eigengenes. The red line represents the height of 0.2. (c) Visualization of GCMs before and after merging closer GCMs according to the height of 0.2. Colors indicate GCMs, and leaves represent genes

Coefficient of variation (CV) of every gene between GDM samples at first‐trimester or second‐trimester pregnancy age and control samples was calculated, and expression profiles of overlapping genes between the top 5,000 genes with largest CV in GDM samples at first and second trimester were used for identification of valuable GCMs. As a result, 3,731 genes were screened and sample clustering based on those genes’ Euclidean distance separated samples into two distinct clusters that contained control and GDM samples, respectively, as shown in Figure 1a.

A total of 33 GCMs were identified based on the soft threshold of 11. Module clustering analysis was further performed based on correlations among eigengenes of the 33 GCMs, which produced seven merged GCMs according to height of 0.2 (red line in Figure 1b). Figure 1c illustrated the GCMs before and after merging closer GCMs.

### 3.2 Module‐trait correlation analysis

**Figure 2:**
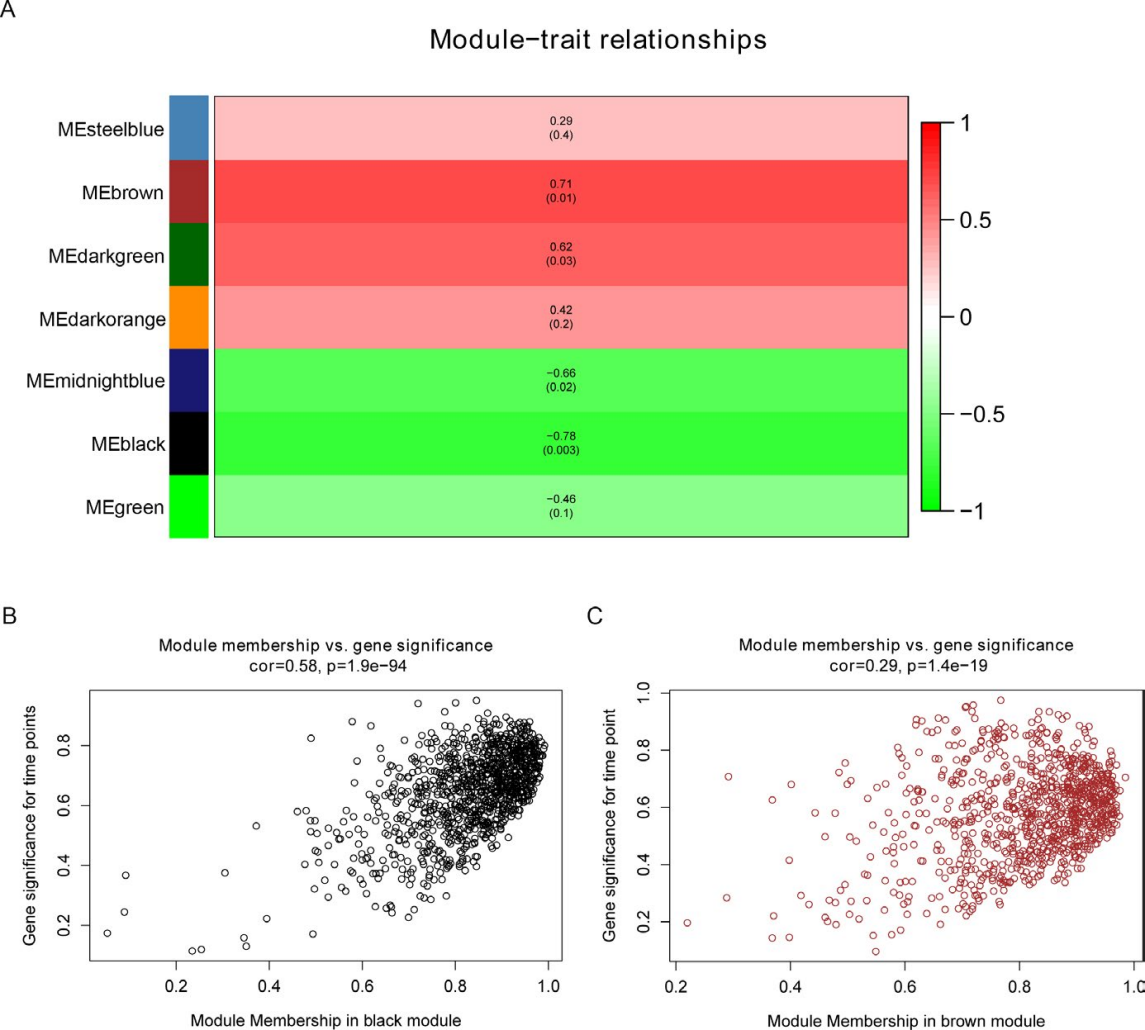
Gene coexpression module (GCM)‐gestational diabetes mellitus (GDM) progression correlation analysis. (a) A heatmap visualization of correlation between GCM and GDM patients’ pregnancy age. Numbers outside and inside brackets represent correlation coefficients and *p* value, respectively. (b,c) was the correlation plot of GS versus module membership for gene contained in black and brown module, respectively

To screen candidate GCMs that might contribute GDM progression, we used GDM patients’ pregnancy age as clinical variable and estimated their correlations with GCMs’ eigengene. As a result, brown and black modules were closely correlated with pregnancy age of GDM patients by using correlation *p* value ≤0.01 as threshold (Figure 2a). Additionally, we calculated correlation between every gene's module membership (MM, correlation between a specific gene and module's eigengene) and gene significance (GS, correlation between a specific gene and clinical variable) in brown and black modules. As expected, significant positive correlations between gene's MM and GS were obtained in black as well as brown module as shown in Figure 2b,c, respectively.

### 3.3 Protein–protein interaction network

**Figure 3:**
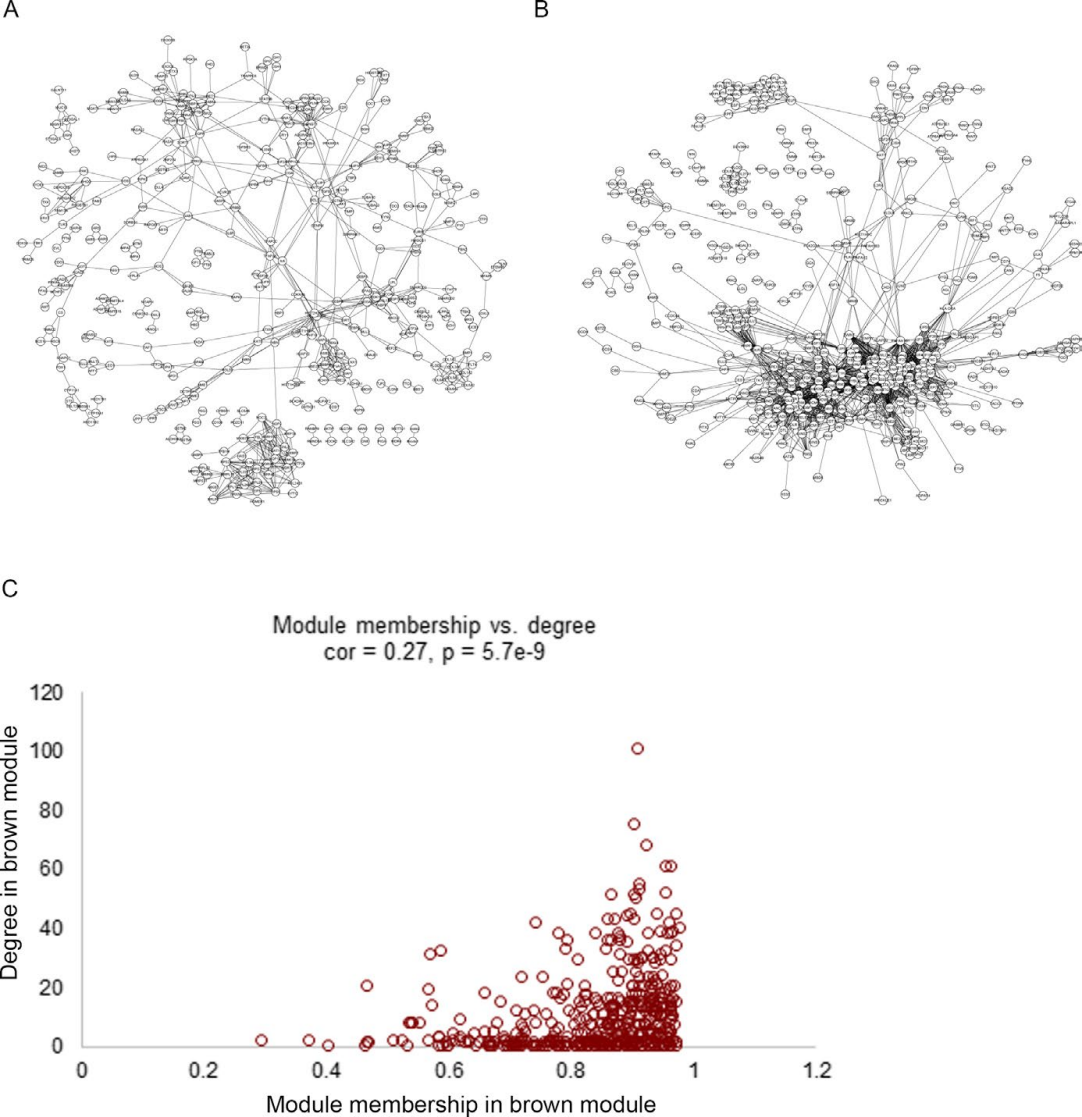
Protein–protein interaction (PPI) network analysis of genes contained in black and brown modules. (a) PPI network of genes contained in the black module. (b) PPI network of genes contained in the brown module. (c) Scatter plot indicated the positive correlation between network degree and module membership of genes contained in the brown module

Identification of GCMs was purely based on mathematical correlations among genes in specific module, so we further explored potential biological associations among genes in brown and black modules. As a result, a total of 842 and 2,654 gene pairs were, respectively, obtained for genes contained in black and brown module. Figure 3a,b illustrate PPI network that comprised of pairs among genes in black and brown module, respectively. Additionally, degree of brown module genes (direct neighborhood in PPI network) was significantly positively correlated with their MM (Figure 3c), which might indicate closer relation between brown module and GDM progression than that of black module. Table 1 shows the genes that with PPI degree larger than 50 and their MM.

**Table 1:** Module membership (MM) and degree of genes that with degree larger than 10 in protein–protein interaction network

Gene	MM	Degree	Gene	MM	Degree
CDK1	0.90	102	AURKB	0.91	54
CCNB1	0.90	76	CCNA2	0.95	53
PLK1	0.92	69	CENPE	0.90	52
CCNB2	0.96	62	PAFAH1B1	0.86	52
CDC20	0.95	62	KIF2C	0.90	51
MAD2L1	0.91	56			

### 3.4 Gene set enrichment analysis

**Figure 4:**
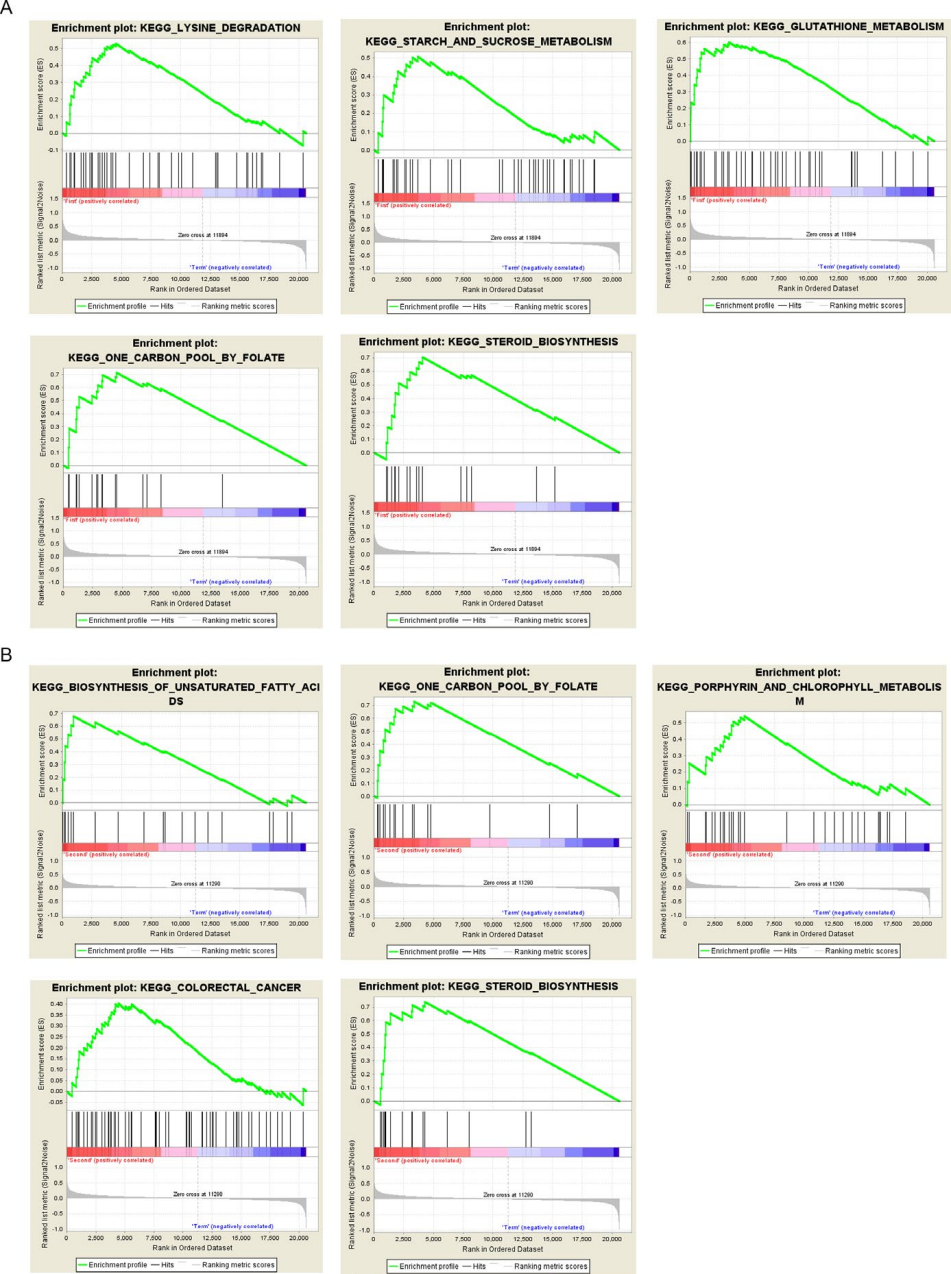
Gene set enrichment analysis. (a) The full list of significantly up‐regulated KEGG pathways in first‐trimester gestational diabetes mellitus (GDM) samples compared with normal samples. (b) The top five most significantly up‐regulated KEGG pathways in second‐trimester GDM samples compared with normal samples

We divided samples into first trimester and control group or second trimester and control group and subjected them to GSEA. Figure 4a,b illustrated the full list of KEGG pathways and top five KEGG pathways that significantly up‐regulated in first‐trimester and second‐trimester GDM samples compared with control samples, respectively. Table 2 is the full list of KEGG pathways significantly up‐regulated in second‐trimester GDM samples. Lysine degradation, carbon pool by folate, and steroid biosynthesis pathways were found to be significantly up‐regulated in both first‐trimester and second‐trimester GDM samples. Strikingly, cancer‐related pathways, such as colorectal cancer, cell cycle, were also significantly up‐regulated in second‐trimester GDM samples.

**Table 2:** Full list of KEGG pathways that significantly up‐regulated in second‐trimester gestational diabetes mellitus samples

Pathway name	ES	Nom *p* value
KEGG_BIOSYNTHESIS_OF_UNSATURATED_FATTY_ACIDS	0.677	0
KEGG_ONE_CARBON_POOL_BY_FOLATE	0.727	0
KEGG_PORPHYRIN_AND_CHLOROPHYLL_METABOLISM	0.539	0
KEGG_COLORECTAL_CANCER	0.404	0
KEGG_STEROID_BIOSYNTHESIS	0.738	0.0192
KEGG_LYSINE_DEGRADATION	0.556	0.0195
KEGG_GLYCEROLIPID_METABOLISM	0.417	0.0200
KEGG_CELL_CYCLE	0.577	0.0303
KEGG_AMINOACYL_TRNA_BIOSYNTHESIS	0.567	0.0306
KEGG_VIBRIO_CHOLERAE_INFECTION	0.412	0.0446
KEGG_RNA_POLYMERASE	0.460	0.0497

Note

ES: enrichment score; Nom *p* value: normalized *p* value.

## 4 DISCUSSION

Gestational diabetes mellitus represents the most prevalence form of pathoglycemia in pregnancy that deeply affects the life of mothers as well as their offspring. In this study, we identified some potential GDM‐related genes by analyzing GDM time series gene expression profiles through WGCNA along with PPI network analysis. GSEA strikingly obtained some cancer‐related pathways in addition to several well‐known GDM‐associated pathways which were significantly up‐regulated in GDM samples compared with control samples. This study should shed some new light on the understanding of GDM mechanisms and its diagnosis or treatment.

Abnormal insulin secretion and metabolism contribute greatly to GDM initiation and progression. Here, lysine degradation, carbon pool by folate, and steroid biosynthesis pathways were significantly up‐regulated in first as well as second‐trimester GDM samples compared with control samples. Lysine represents a major component of histone, and modifications such as acetylation, methylation, and so on in it play vital roles in major cellular functions, for example, posttranscriptional proteins’ modification. In addition, lysine acetylation was also previously reported to affect both immunological and metabolic pathways, which could then induce type II diabetes and cardiovascular disease (Iyer & Fairlie, 2012; Kosanam et al., 2014). Nε‐(carboxymethyl) lysine‐conjugated bovine serum albumin is an essential component of advanced glycation end products which could damage mitochondrial functions and result in reducing insulin secretion followed by the incidence of diabetes (Lo et al., 2015). Diabetes could control many aspects of endocrine, including steroidogenesis, whose perturbation could in turn induce the initiation of diabetes (Hwang, 2014; Jangir, 2014). In addition to some well‐known pathways related to GDM, we strikingly identified the up‐regulation of several cancer‐related pathways in second‐trimester GDM samples, such as colorectal cancer, cell cycle. It has been widely reported that diabetes could result in elevated colorectal cancer risk (Jarvandi & Davidson, 2013; Wu et al., 2013), which was also confirmed in a rat model (Jia et al., 2014).

MM and degree are important for evaluating genes’ associations with specific trait in gene coexpression and PPI network, respectively. In this study, several genes have both high MM in brown module and high degree in PPI network, which might serve as important diagnosis or treatment biomarkers for GDM. *CDK1* and *CCNB1* are the top two genes with larger degree in PPI network, and *CDK1* and *CCNB1* interaction was previously proved to coordinates mitochondrial respiration and affect G2/M cell cycle progression (Wang et al., 2014). Cell cycle perturbation is closely associated with multi diseases’ progression including diabetes (Saavedra‐Avila et al., 2014; Wang, Fiaschi‐Taesch, et al., 2015). Besides, aberrant expression of *CDK1* and *CCNB1* in diabetes patients were also proved by previous studies (Page, Morris, Williams, Ruhland, & Malik, 1997; Su et al., 2015). *CCNB2*’s MM is the largest one compared with the other nine genes in Table 1. In Zhang's study (2017), *CCNB2* was found to be significantly up‐regulated in diabetes mice compared with normal mice and was also the core gene in PPI network of DEGs. Although no previous study about direct association between some of those genes with GDM progression, lots of studies have proved associations between those genes with the well‐known GDM‐associated biological processes, such as *CDC20* with metabolism (Martin, Mebarki, Paradis, Friguet, & Radman, 2014), *CENPE* with insulin absorption (Zhu, Ai, Wang, Xu, & Teng, 2012), and so on. So, those genes might be potential biomarkers for GDM.

## 5 CONCLUSIONS

In conclusion, we identified several potential biomarkers for GDM through WGCNA and PPI network analysis. GSEA identified some cancer‐related pathways in addition to several well‐known GDM‐related pathways, which might provide novel clues for GDM experimental research and clinical treatment.

## CONFLICT OF INTEREST

I declare that there is no conflict between authors.
